# {*N* ^1^-[2-(Butyl­selan­yl)benz­yl]-*N* ^2^,*N* ^2^-di­methyl­ethane-1,2-di­amine}­dichlorido­mercury(II)

**DOI:** 10.1107/S2056989018010423

**Published:** 2018-07-27

**Authors:** Pushpendra Singh, Harkesh B. Singh, Ray J. Butcher

**Affiliations:** a Department of Chemistry, Dr. Shakuntala Misra National Rehabilitation University, Mohaan Road Lucknow, 226017, India; b Department of Chemistry, Indian Institute of Technology Bombay, Powai 400 076, Mumbai, India; c Department of Chemistry, Howard University, 525 College Street NW, Washington, DC 20059, USA

**Keywords:** crystal structure, mercury (II) complexes, mercury–selenium bonding

## Abstract

In the title compound, [HgCl_2_(C_16_H_28_N_2_Se)], the primary geometry around the Se and Hg atoms is distorted trigonal–pyramidal and distorted square-pyramidal, respectively. The distortion of the mol­ecular geometry in the complex is caused by the steric demands of the ligands attached to the Se atom. The Hg atom is coordinated through two chloride anions, an N atom and an Se atom, making up an unusual HgNSeCl_2_ coordination sphere with an additional long Hg⋯N inter­action. Inter­molecular C—H⋯Cl inter­actions are the only identified inter­molecular hydrogen-bonding inter­actions that seem to be responsible for the self assembly. These relatively weak C—H⋯Cl hydrogen bonds possess the required linearity and donor–acceptor distances. They act as mol­ecular associative forces that result in a supra­molecular assembly along the *b-*axis direction in the solid state of the title compound.

## Abstract 2

In the structure of C_16_H_28_N_2_SeHgCl_2_ the mercury atom is coordinated through two chloride anions, a nitro­gen atom and a selenium atom to make up an unusual HgNSeCl_2_ coordination sphere with an additional long Hg⋯N inter­action.

## Chemical context   

The chemistry of mercuric compounds with multidentate amine ligands is of inter­est because of the low coordination number and geometry preferences of the Hg^II^ atom, which facilitates extraordinarily rapid exchange of simple ligands (Bebout *et al.*, 2013 ▸; Carra *et al.*, 2013 ▸). The enhanced binding thermodynamics of these multidentate ligands has been used to suppress inter­molecular ligand-exchange rates for a variety of Hg^II^ complexes in solution, greatly enhancing the meaningfulness of NMR characterization. Significantly, under conditions of slow inter­molecular exchange, the rates of intra­molecular isomerization processes for Hg^II^ can still exceed both the chemical shift and coupling constant time scale, particularly when bond cleavage is unnecessary and the structures of these complexes have been determined (Bebout *et al.*, 2013 ▸; Carra *et al.*, 2013 ▸).
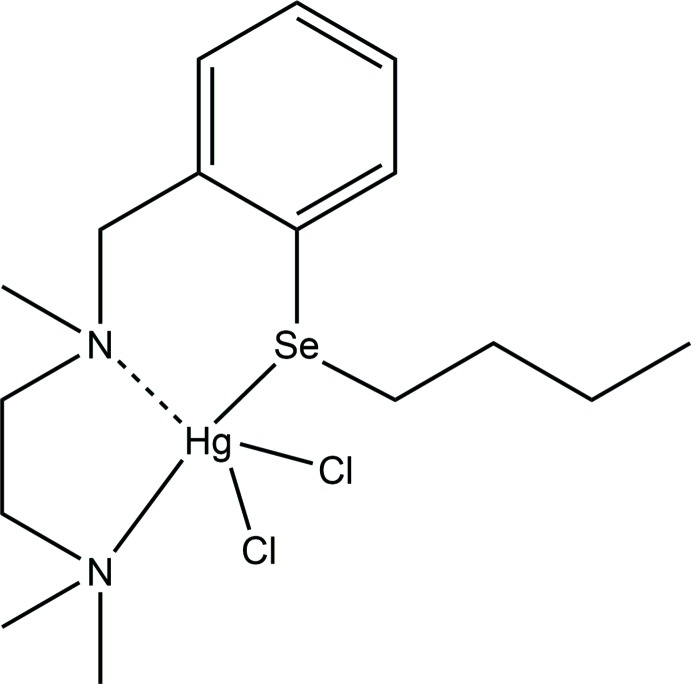


As part of our continuing studies in this area, we have been investigating the structural chemistry of mercuric compounds with multidentate amine ligands combined with either Se (Manjare *et al.*, 2014 ▸) or Te (Singh *et al.*, 2003 ▸) as an additional ligand in the presence of an Hg*X*_2_ group (*X* = Cl, Br, or I) and the structure of the title compound is reported herein.

## Structural commentary   

**Figure 1:**
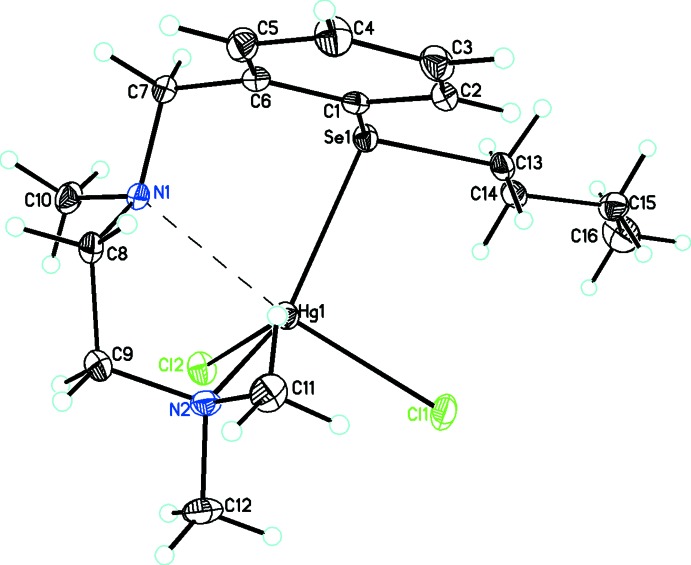
The mol­ecular structure of {*N* ^1^-[2-(Butyl­selan­yl)benz­yl]-*N* ^2^,*N* ^2^-di­methyl­ethane-1,2-di­amine}­dichlorido­mercury(II). The inter­action between Hg1 and N1 is shown with a dashed line. Anisotropic displacement parameters are at the 30% probability level.

The title compound, C_16_H_28_N_2_SeHgCl_2_, crystallizes in the monoclinic crystal system and the mol­ecular structure is shown in Fig. 1 ▸. The primary geometry around the Se and Hg atoms of [2-{Me_2_NCH_2_CH_2_N(Me)}C_6_H_4_SeBu]HgCl_2_ is distorted trigonal–pyramidal and distorted square-pyramidal, respectively. The distortion of the mol­ecular geometry in the complex is caused by the steric demands of the ligands attached to the selenium atom. The mercury atom is coordinated through two chloride anions, a nitro­gen atom and a selenium atom to make up an unusual HgNSeCl_2_ coordination sphere. In this complex the 2-{Me_2_NCH_2_CH_2_N(Me)}C_6_H_4_SeBu ligand is acting in a bidentate fashion, leading to the formation of a nine-membered chelate ring. There is only one such example in the Cambridge Structural Database (CSD Version 5.39, November 2017 update; Groom *et al.*, 2016 ▸) of an HgCl_2_ complex containing a similar set of coordinated donor atoms (Apte *et al.*, 2003 ▸). In addition to the coordinated atoms, there is an inter­action between Hg and N1 [2.712 (2) Å; Table 1 ▸] that is greater than Σ*r*_cov_ (Hg,N), 2.03 Å, but significantly shorter than Σ*r*_vdw_ (Hg,N), 3.53 Å and indicates the presence of an attractive N⋯Hg inter­action (Bondi, 1964 ▸; Canty & Deacon, 1980 ▸; Pyykkö & Straka, 2000 ▸; Batsanov, 2001 ▸); this is clearly shown in Fig. 1 ▸, where the ligand has adopted a conformation which brings N1 close to Hg1.

In the title complex, the Hg—Cl distances, 2.4515 (7) and 2.5380 (8) Å, are in the normal range for such distances [a survey of the CSD for N–Hg–Cl complexes gave 87 hits with a mean Hg—Cl distance of 2.45 (18) Å], while the Hg—N2 distance is 2.359 (2) Å, which is shorter than the mean value for such distances [a survey of the CSD for Cl–Hg–N compounds gave 82 hits with a mean Hg—N distance of 2.50 (16) Å]. A related HgCl_2_ complex with a similar ligand but without the *n*-butyl­selenium substituent has been reported [*N* ^1^-benzyl-*N* ^1^,*N* ^2^,*N* ^2^-tri­methyl­ethane-1,2-di­amine; Manjare *et al.*, 2014 ▸] in which the Hg atom is coordinated to both N donors with Hg—N distances of 2.355 (4) and 2.411 (4) Å. The Hg—Se distance of 2.6950 (3) Å in the title compound is in the normal range [a survey of the CSD for phen­yl–Hg–Se compounds gave 82 hits with a mean Hg—Se distance of 2.67 (11) Å] and is close to Σ*r*_co__v_ (Se—Hg), 2.52 Å and much smaller than the Σ*r*_vdw_ (3.88 Å), thus indicating the presence of a very strong Se—Hg inter­action (Bondi, 1964 ▸; Canty & Deacon, 1980 ▸; Pyykkö & Straka, 2000 ▸; Batsanov, 2001 ▸). This bond length is close that observed in [C_6_H_4_(C_5_H_8_NO)]_2_SeHgCl_2_ [2.750 (7) Å; Apte *et al.*, 2003 ▸] but is longer than the reported value in the tetra­hedral complex of an Hg seleno­phene, HgBr_2_(C_4_H_8_Se)_2_ [2.648 (1) Å; Stålhandske & Zintl, 1988 ▸].

## Supra­molecular features   

**Figure 2:**
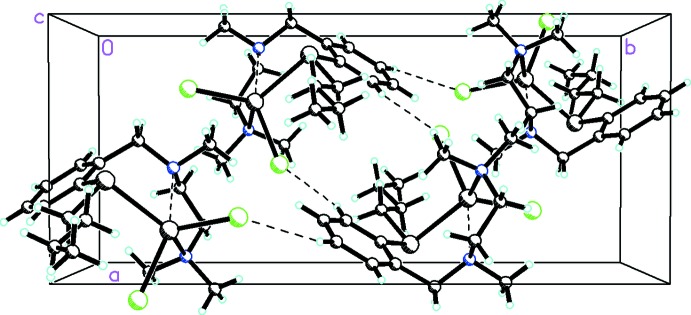
Packing diagram of the title compound viewed along the *c* axis showing how the C—H⋯Cl inter­actions (shown with dashed lines) link the mol­ecules into chains along the *b-*axis direction.

Inter­molecular C—H⋯Cl inter­actions (Table 2 ▸, Fig. 2 ▸) are the only identified inter­molecular hydrogen-bonding inter­action that seems to be responsible for the self-assembly. These relatively weak C—H⋯Cl hydrogen bonds possess the required linearity and donor–acceptor distances. They act as mol­ecular associative forces that result in a supra­molecular assembly along the *b*-axis direction.

## Database survey   

There is only one such example of an HgCl_2_ complex containing a similar set of coordinated donor atoms in the CSD [Version 5.39, November 2017 update; Groom *et al.*, 2016 ▸] *viz*. ERIBAI (Apte *et al.*, 2003 ▸).

## Synthesis and crystallization   

**Synthesis of 2-{Me_2_NCH_2_CH_2_N(Me)}C_6_H_4_Se(** ***n*** **-but­yl)**

The 2-{Me_2_NCH_2_CH_2_N(Me)}C_6_H_4_Br ligand was prepared by following the reported procedure (Rietveld *et al.*, 1994 ▸). A stirred solution of 2-{Me_2_NCH_2_CH_2_N(Me)}C_6_H_4_Br (1.10 ml, 5.34 mmol) in dry THF (15 mL) was treated dropwise with an 1.6 *M* solution of *n*-BuLi in hexane (6.20 mL, 10.0 mmol) *via* syringe under N_2_ at 273 K. After stirring the reaction mixture for 2 h at this temperature, the li­thia­ted product was obtained. Selenium powder (0.45 g, 5.70 mmol) was added to the solution under a brisk flow of N_2_ gas and stirring was continued for an additional 2 h at 273 K. The reaction mixture was then removed from the N_2_ line and poured into a beaker containing water. The organic phase was separated, dried over Na_2_SO_4_, and filtered. The filtrate was evaporated to dryness to give a yellow oil of 2-{Me_2_NCH_2_CH_2_N(Me)}C_6_H_4_Se(*n*-but­yl). The product was used as such without further purification. ^77^Se NMR (76.3 MHz, CDCl_3_) *δ* 247.5.

**Synthesis of [2-{Me_2_NCH_2_CH_2_N(Me)}C_6_H_4_Se** ***^n^*** **Bu]HgCl_2_**

To a 50 mL two-necked flask, was taken a chloro­form solution (7 mL) of 2-{Me_2_NCH_2_CH_2_N(Me)}C_6_H_4_Se(*n*-but­yl) (0.51 g, 1.56 mmol). To it was added an aceto­nitrile solution (5 mL) of HgCl_2_ (0.43 g, 1.56 mmol). The mixture was stirred for 1 h to obtain a white precipitate, which was recrystallized from chloro­form to give [2-{Me_2_NCH_2_CH_2_N(Me)}C_6_H_4_Se^*n*^Bu]HgCl_2_ (0.52 g, 55% yield), m.p. 431 K. ^1^H NMR (400 MHz, CDCl_3_) *δ* 0.95 (*t*, *J* = 7.0 Hz, 3H), 1.50 (*sextet*, *J* = 7.0 and 8.0 Hz, 2H), 1.80 (*quintet*, *J* = 7.0 and 8.0 Hz, 2H), 2.13 (*s*, *br*, NCH_3_), 2.49 (*s*, N(CH_3_)_2_), 3.38 (*s*, *br*, 2H), 3.76 (*s*, *br*, 2H), 7.24–7.36 (*m*, 3H-ar­yl), 7.46 (*b*, *J* = 7.6 Hz, 1H-ar­yl); ^13^C NMR (100.6 MHz, CDCl_3_) *δ* 13.8, 23.2, 29.4, 30.5, 43.9, 52.5, 56.5, 63.6, 127.5, 126.4, 129.7, 131.3, 131.8, 136.0; ^77^Se NMR (76.3 MHz, CDCl_3_) *δ* 223.6. Anaysis calculated for C_16_H_28_N_2_SeHgCl_2_: C, 32.09; N, 4.68; H, 4.71. Found C, 31.49; N, 4.98; H, 4.19.

## Refinement   

Crystal data, data collection and structure refinement details are summarized in Table 3 ▸. The H atoms were positioned geometrically and allowed to ride on their parent atoms, with C—H = ranging from 0.95 to 0.99 Å and *U*_iso_(H) = *xU*_eq_(C), where *x* = 1.5 for methyl H atoms and 1.2 for all other C-bound H atoms.

## Supplementary Material

Crystal structure: contains datablock(s) I. DOI: 10.1107/S2056989018010423/lh5877sup1.cif

Structure factors: contains datablock(s) I. DOI: 10.1107/S2056989018010423/lh5877Isup2.hkl

CCDC reference: 1814426

Additional supporting information:  crystallographic information; 3D view; checkCIF report

## Figures and Tables

**Table 1:** Selected geometric parameters (Å, °)

Se1—C1	1.925 (3)	Hg1—Cl2	2.4515 (7)
Se1—C13	1.956 (3)	Hg1—Cl1	2.5380 (8)
Se1—Hg1	2.6950 (3)	Hg1—N1	2.712 (2)
Hg1—N2	2.359 (2)		
			
C1—Se1—C13	101.89 (13)	Cl2—Hg1—Se1	116.79 (2)
C1—Se1—Hg1	93.12 (8)	Cl1—Hg1—Se1	97.86 (2)
C13—Se1—Hg1	103.00 (9)	N2—Hg1—N1	71.81 (8)
N2—Hg1—Cl2	103.62 (6)	Cl2—Hg1—N1	96.11 (5)
N2—Hg1—Cl1	91.40 (7)	Cl1—Hg1—N1	150.49 (5)
Cl2—Hg1—Cl1	111.65 (3)	Se1—Hg1—N1	77.25 (5)
N2—Hg1—Se1	131.01 (6)		

**Table 2:** Hydrogen-bond geometry (Å, °)

*D*—H⋯*A*	*D*—H	H⋯*A*	*D*⋯*A*	*D*—H⋯*A*
C2—H2*A*⋯Cl1^i^	0.95	2.71	3.538 (3)	146
C11—H11*A*⋯Cl2^ii^	0.98	2.83	3.726 (3)	152
C11—H11*C*⋯Cl1	0.98	2.92	3.576 (4)	125
C12—H12*B*⋯Cl1	0.98	2.98	3.636 (4)	125
C13—H13*B*⋯Cl1	0.99	2.85	3.560 (3)	130

Symmetry codes: (i) 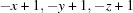; (ii) 
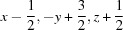
.

**Table 3:** Experimental details

Crystal data	
Chemical formula	[HgCl_2_(C_16_H_28_N_2_Se)]
*M*_r_	598.85
Crystal system, space group	Monoclinic, *P*2_1_/*n*
Temperature (K)	173
*a*, *b*, *c* (Å)	8.5532 (1), 19.6993 (3), 11.9128 (2)
β (°)	91.935 (1)
*V* (Å^3^)	2006.07 (5)
*Z*	4
Radiation type	Mo *K*α
μ (mm^−1^)	9.75
Crystal size (mm)	0.14 × 0.12 × 0.10

Data collection	
Diffractometer	Oxford Diffraction Xcalibur Eos Gemini
Absorption correction	Multi-scan (*CrysAlis RED*; Oxford Diffraction, 2010 ▸)
*T*_min_, *T*_max_	0.342, 0.442
No. of measured, independent and observed [*I* > 2σ(*I*)] reflections	18326, 5418, 4692
*R*_int_	0.035
(sin θ/λ)_max_ (Å^−1^)	0.709

Refinement	
*R*[*F* ^2^ > 2σ(*F* ^2^)], *wR*(*F* ^2^), *S*	0.024, 0.048, 1.05
No. of reflections	5418
No. of parameters	204
H-atom treatment	H-atom parameters constrained
Δρ_max_, Δρ_min_ (e Å^−3^)	0.76, −0.68

Computer programs: *CrysAlis PRO* and *CrysAlis RED* (Oxford Diffraction, 2010 ▸), *SHELXS97* and *SHELXTL* (Sheldrick, 2008 ▸) and *SHELXL2018* (Sheldrick, 2015 ▸).

## supplementary crystallographic information

### Crystal data

**Table:**

[HgCl_2_(C_16_H_28_N_2_Se)]	*F*(000) = 1144
*M**_r_* = 598.85	*D*_x_ = 1.983 Mg m^−^^3^
Monoclinic, *P*2_1_/*n*	Mo *K*α radiation, λ = 0.71073 Å
*a* = 8.5532 (1) Å	Cell parameters from 9237 reflections
*b* = 19.6993 (3) Å	θ = 3.1–30.2°
*c* = 11.9128 (2) Å	µ = 9.75 mm^−^^1^
β = 91.935 (1)°	*T* = 173 K
*V* = 2006.07 (5) Å^3^	Block, colorless
*Z* = 4	0.14 × 0.12 × 0.10 mm

### Data collection

**Table:**

Oxford Diffraction Xcalibur Eos Gemini diffractometer	4692 reflections with *I* > 2σ(*I*)
Detector resolution: 16.1500 pixels mm^-1^	*R*_int_ = 0.035
ω scans	θ_max_ = 30.3°, θ_min_ = 3.1°
Absorption correction: multi-scan (CrysAlis RED; Oxford Diffraction, 2010)	*h* = −11→11
*T*_min_ = 0.342, *T*_max_ = 0.442	*k* = −25→27
18326 measured reflections	*l* = −16→16
5418 independent reflections	

### Refinement

**Table:**

Refinement on *F*^2^	Secondary atom site location: difference Fourier map
Least-squares matrix: full	Hydrogen site location: inferred from neighbouring sites
*R*[*F*^2^ > 2σ(*F*^2^)] = 0.024	H-atom parameters constrained
*wR*(*F*^2^) = 0.048	*w* = 1/[σ^2^(*F*_o_^2^) + (0.0172*P*)^2^] where *P* = (*F*_o_^2^ + 2*F*_c_^2^)/3
*S* = 1.05	(Δ/σ)_max_ = 0.004
5418 reflections	Δρ_max_ = 0.76 e Å^−^^3^
204 parameters	Δρ_min_ = −0.68 e Å^−^^3^
0 restraints	Extinction correction: SHELXL2018 (Sheldrick, 2015), Fc^*^=kFc[1+0.001xFc^2^λ^3^/sin(2θ)]^-1/4^
Primary atom site location: structure-invariant direct methods	Extinction coefficient: 0.00039 (7)

### Special details

**Table:**

Geometry. All esds (except the esd in the dihedral angle between two l.s. planes) are estimated using the full covariance matrix. The cell esds are taken into account individually in the estimation of esds in distances, angles and torsion angles; correlations between esds in cell parameters are only used when they are defined by crystal symmetry. An approximate (isotropic) treatment of cell esds is used for estimating esds involving l.s. planes.

### Fractional atomic coordinates and isotropic or equivalent isotropic displacement parameters (Å^2^)

**Table:**

	*x*	*y*	*z*	*U*_iso_*/*U*_eq_	
Se1	0.88460 (3)	0.58884 (2)	0.51900 (3)	0.02289 (7)	
Hg1	0.69533 (2)	0.68781 (2)	0.59155 (2)	0.02300 (5)	
Cl1	0.43933 (9)	0.64178 (4)	0.50887 (8)	0.0387 (2)	
Cl2	0.74703 (10)	0.80320 (4)	0.52526 (7)	0.03128 (18)	
N1	0.9280 (3)	0.68551 (11)	0.7519 (2)	0.0206 (5)	
N2	0.5831 (3)	0.70509 (12)	0.7674 (2)	0.0240 (5)	
C1	0.8665 (3)	0.53631 (14)	0.6542 (2)	0.0205 (6)	
C2	0.7814 (3)	0.47607 (15)	0.6550 (3)	0.0252 (6)	
H2A	0.728128	0.460499	0.588628	0.030*	
C3	0.7754 (4)	0.43925 (15)	0.7532 (3)	0.0304 (7)	
H3A	0.716904	0.398275	0.754398	0.036*	
C4	0.8531 (4)	0.46117 (16)	0.8497 (3)	0.0331 (8)	
H4A	0.848444	0.435399	0.916944	0.040*	
C5	0.9380 (4)	0.52084 (16)	0.8482 (3)	0.0291 (7)	
H5A	0.992037	0.535596	0.914759	0.035*	
C6	0.9457 (3)	0.55990 (14)	0.7506 (3)	0.0214 (6)	
C7	1.0347 (3)	0.62626 (14)	0.7524 (3)	0.0225 (6)	
H7A	1.104342	0.627991	0.820418	0.027*	
H7B	1.100979	0.628575	0.685954	0.027*	
C8	0.8476 (3)	0.69056 (15)	0.8591 (3)	0.0244 (6)	
H8A	0.919412	0.711348	0.916250	0.029*	
H8B	0.821384	0.644354	0.885204	0.029*	
C9	0.6990 (3)	0.73248 (16)	0.8492 (3)	0.0276 (7)	
H9A	0.651544	0.734828	0.923782	0.033*	
H9B	0.726285	0.779307	0.826940	0.033*	
C10	1.0194 (3)	0.74751 (15)	0.7331 (3)	0.0297 (7)	
H10A	0.950050	0.787094	0.734383	0.045*	
H10B	1.068037	0.744760	0.659927	0.045*	
H10C	1.100872	0.751967	0.792440	0.045*	
C11	0.5182 (4)	0.64009 (16)	0.8056 (3)	0.0340 (8)	
H11A	0.474667	0.646063	0.879989	0.051*	
H11B	0.601415	0.605887	0.809845	0.051*	
H11C	0.435483	0.625092	0.752337	0.051*	
C12	0.4560 (4)	0.75489 (18)	0.7502 (3)	0.0379 (8)	
H12A	0.408771	0.764699	0.822197	0.057*	
H12B	0.376367	0.736263	0.697739	0.057*	
H12C	0.498469	0.796830	0.719106	0.057*	
C13	0.7492 (4)	0.53852 (15)	0.4128 (3)	0.0255 (6)	
H13A	0.789231	0.491767	0.403698	0.031*	
H13B	0.641841	0.535808	0.441121	0.031*	
C14	0.7473 (4)	0.57511 (15)	0.3018 (3)	0.0288 (7)	
H14A	0.856197	0.582261	0.278803	0.035*	
H14B	0.698200	0.620225	0.310579	0.035*	
C15	0.6578 (4)	0.53556 (17)	0.2105 (3)	0.0321 (7)	
H15A	0.708908	0.490984	0.200433	0.038*	
H15B	0.549999	0.527132	0.234860	0.038*	
C16	0.6505 (5)	0.5727 (2)	0.0989 (3)	0.0530 (11)	
H16A	0.588490	0.546106	0.043971	0.080*	
H16B	0.756675	0.578695	0.072075	0.080*	
H16C	0.601684	0.617197	0.108610	0.080*	

### Atomic displacement parameters (Å^2^)

**Table:**

	*U*^11^	*U*^22^	*U*^33^	*U*^12^	*U*^13^	*U*^23^
Se1	0.02570 (15)	0.02228 (15)	0.02057 (16)	−0.00100 (11)	−0.00104 (12)	−0.00145 (12)
Hg1	0.02578 (7)	0.01907 (6)	0.02407 (7)	−0.00076 (4)	−0.00037 (5)	−0.00041 (5)
Cl1	0.0347 (4)	0.0346 (4)	0.0457 (5)	−0.0054 (3)	−0.0151 (4)	−0.0067 (4)
Cl2	0.0425 (5)	0.0220 (4)	0.0292 (4)	−0.0023 (3)	−0.0005 (4)	0.0049 (3)
N1	0.0184 (11)	0.0194 (12)	0.0237 (14)	−0.0023 (9)	−0.0028 (10)	−0.0028 (10)
N2	0.0185 (12)	0.0240 (13)	0.0296 (15)	0.0016 (10)	0.0031 (11)	0.0002 (11)
C1	0.0192 (14)	0.0190 (14)	0.0232 (15)	0.0049 (11)	−0.0003 (12)	−0.0008 (12)
C2	0.0263 (15)	0.0208 (15)	0.0283 (17)	0.0014 (12)	−0.0025 (13)	−0.0051 (13)
C3	0.0333 (17)	0.0203 (15)	0.038 (2)	−0.0031 (13)	0.0034 (15)	0.0016 (14)
C4	0.044 (2)	0.0263 (16)	0.0286 (18)	−0.0018 (14)	−0.0015 (16)	0.0076 (14)
C5	0.0340 (17)	0.0264 (16)	0.0266 (17)	0.0014 (13)	−0.0071 (14)	0.0005 (13)
C6	0.0187 (13)	0.0210 (14)	0.0244 (16)	0.0032 (11)	−0.0009 (12)	−0.0008 (12)
C7	0.0206 (14)	0.0240 (15)	0.0227 (16)	−0.0004 (11)	−0.0032 (12)	−0.0036 (12)
C8	0.0264 (15)	0.0252 (15)	0.0213 (16)	−0.0016 (12)	−0.0038 (13)	−0.0049 (13)
C9	0.0286 (16)	0.0284 (16)	0.0258 (17)	0.0008 (13)	0.0010 (13)	−0.0075 (13)
C10	0.0253 (16)	0.0266 (16)	0.0367 (19)	−0.0077 (13)	−0.0046 (14)	−0.0003 (14)
C11	0.0311 (17)	0.0340 (18)	0.037 (2)	−0.0088 (14)	0.0084 (15)	0.0047 (15)
C12	0.0229 (16)	0.041 (2)	0.050 (2)	0.0099 (14)	0.0028 (15)	0.0018 (17)
C13	0.0299 (16)	0.0234 (15)	0.0231 (16)	0.0015 (13)	−0.0024 (13)	−0.0067 (13)
C14	0.0330 (17)	0.0224 (15)	0.0307 (18)	0.0026 (13)	−0.0009 (14)	−0.0036 (14)
C15	0.0317 (17)	0.0388 (19)	0.0254 (18)	0.0045 (14)	−0.0031 (14)	−0.0016 (15)
C16	0.075 (3)	0.057 (3)	0.026 (2)	0.015 (2)	−0.012 (2)	−0.0016 (18)

### Geometric parameters (Å, º)

**Table:**

Se1—C1	1.925 (3)	C8—C9	1.517 (4)
Se1—C13	1.956 (3)	C8—H8A	0.9900
Se1—Hg1	2.6950 (3)	C8—H8B	0.9900
Hg1—N2	2.359 (2)	C9—H9A	0.9900
Hg1—Cl2	2.4515 (7)	C9—H9B	0.9900
Hg1—Cl1	2.5380 (8)	C10—H10A	0.9800
Hg1—N1	2.712 (2)	C10—H10B	0.9800
N1—C10	1.471 (3)	C10—H10C	0.9800
N1—C8	1.474 (4)	C11—H11A	0.9800
N1—C7	1.482 (3)	C11—H11B	0.9800
N2—C9	1.470 (4)	C11—H11C	0.9800
N2—C12	1.473 (4)	C12—H12A	0.9800
N2—C11	1.473 (4)	C12—H12B	0.9800
C1—C2	1.392 (4)	C12—H12C	0.9800
C1—C6	1.393 (4)	C13—C14	1.505 (4)
C2—C3	1.379 (4)	C13—H13A	0.9900
C2—H2A	0.9500	C13—H13B	0.9900
C3—C4	1.378 (5)	C14—C15	1.523 (5)
C3—H3A	0.9500	C14—H14A	0.9900
C4—C5	1.382 (4)	C14—H14B	0.9900
C4—H4A	0.9500	C15—C16	1.516 (5)
C5—C6	1.398 (4)	C15—H15A	0.9900
C5—H5A	0.9500	C15—H15B	0.9900
C6—C7	1.512 (4)	C16—H16A	0.9800
C7—H7A	0.9900	C16—H16B	0.9800
C7—H7B	0.9900	C16—H16C	0.9800
			
C1—Se1—C13	101.89 (13)	C9—C8—H8B	109.1
C1—Se1—Hg1	93.12 (8)	H8A—C8—H8B	107.8
C13—Se1—Hg1	103.00 (9)	N2—C9—C8	113.4 (2)
N2—Hg1—Cl2	103.62 (6)	N2—C9—H9A	108.9
N2—Hg1—Cl1	91.40 (7)	C8—C9—H9A	108.9
Cl2—Hg1—Cl1	111.65 (3)	N2—C9—H9B	108.9
N2—Hg1—Se1	131.01 (6)	C8—C9—H9B	108.9
Cl2—Hg1—Se1	116.79 (2)	H9A—C9—H9B	107.7
Cl1—Hg1—Se1	97.86 (2)	N1—C10—H10A	109.5
N2—Hg1—N1	71.81 (8)	N1—C10—H10B	109.5
Cl2—Hg1—N1	96.11 (5)	H10A—C10—H10B	109.5
Cl1—Hg1—N1	150.49 (5)	N1—C10—H10C	109.5
Se1—Hg1—N1	77.25 (5)	H10A—C10—H10C	109.5
C10—N1—C8	110.0 (2)	H10B—C10—H10C	109.5
C10—N1—C7	108.9 (2)	N2—C11—H11A	109.5
C8—N1—C7	110.8 (2)	N2—C11—H11B	109.5
C9—N2—C12	109.0 (2)	H11A—C11—H11B	109.5
C9—N2—C11	111.5 (3)	N2—C11—H11C	109.5
C12—N2—C11	109.8 (2)	H11A—C11—H11C	109.5
C9—N2—Hg1	110.86 (17)	H11B—C11—H11C	109.5
C12—N2—Hg1	107.0 (2)	N2—C12—H12A	109.5
C11—N2—Hg1	108.51 (19)	N2—C12—H12B	109.5
C2—C1—C6	121.2 (3)	H12A—C12—H12B	109.5
C2—C1—Se1	121.4 (2)	N2—C12—H12C	109.5
C6—C1—Se1	117.4 (2)	H12A—C12—H12C	109.5
C3—C2—C1	119.2 (3)	H12B—C12—H12C	109.5
C3—C2—H2A	120.4	C14—C13—Se1	108.3 (2)
C1—C2—H2A	120.4	C14—C13—H13A	110.0
C4—C3—C2	120.8 (3)	Se1—C13—H13A	110.0
C4—C3—H3A	119.6	C14—C13—H13B	110.0
C2—C3—H3A	119.6	Se1—C13—H13B	110.0
C3—C4—C5	119.6 (3)	H13A—C13—H13B	108.4
C3—C4—H4A	120.2	C13—C14—C15	111.9 (3)
C5—C4—H4A	120.2	C13—C14—H14A	109.2
C4—C5—C6	121.3 (3)	C15—C14—H14A	109.2
C4—C5—H5A	119.4	C13—C14—H14B	109.2
C6—C5—H5A	119.4	C15—C14—H14B	109.2
C1—C6—C5	117.8 (3)	H14A—C14—H14B	107.9
C1—C6—C7	122.0 (3)	C16—C15—C14	112.6 (3)
C5—C6—C7	120.1 (3)	C16—C15—H15A	109.1
N1—C7—C6	111.8 (2)	C14—C15—H15A	109.1
N1—C7—H7A	109.3	C16—C15—H15B	109.1
C6—C7—H7A	109.3	C14—C15—H15B	109.1
N1—C7—H7B	109.3	H15A—C15—H15B	107.8
C6—C7—H7B	109.3	C15—C16—H16A	109.5
H7A—C7—H7B	107.9	C15—C16—H16B	109.5
N1—C8—C9	112.5 (3)	H16A—C16—H16B	109.5
N1—C8—H8A	109.1	C15—C16—H16C	109.5
C9—C8—H8A	109.1	H16A—C16—H16C	109.5
N1—C8—H8B	109.1	H16B—C16—H16C	109.5
			
C6—C1—C2—C3	0.2 (4)	C8—N1—C7—C6	69.1 (3)
Se1—C1—C2—C3	178.2 (2)	C1—C6—C7—N1	73.8 (3)
C1—C2—C3—C4	−0.4 (4)	C5—C6—C7—N1	−104.7 (3)
C2—C3—C4—C5	0.1 (5)	C10—N1—C8—C9	80.3 (3)
C3—C4—C5—C6	0.5 (5)	C7—N1—C8—C9	−159.3 (2)
C2—C1—C6—C5	0.4 (4)	C12—N2—C9—C8	−170.6 (3)
Se1—C1—C6—C5	−177.7 (2)	C11—N2—C9—C8	68.0 (3)
C2—C1—C6—C7	−178.1 (3)	Hg1—N2—C9—C8	−53.0 (3)
Se1—C1—C6—C7	3.7 (3)	N1—C8—C9—N2	59.7 (3)
C4—C5—C6—C1	−0.7 (4)	Se1—C13—C14—C15	174.2 (2)
C4—C5—C6—C7	177.8 (3)	C13—C14—C15—C16	178.3 (3)
C10—N1—C7—C6	−169.9 (2)		

### Hydrogen-bond geometry (Å, º)

**Table:**

*D*—H···*A*	*D*—H	H···*A*	*D*···*A*	*D*—H···*A*
C2—H2*A*···Cl1^i^	0.95	2.71	3.538 (3)	146
C11—H11*A*···Cl2^ii^	0.98	2.83	3.726 (3)	152
C11—H11*C*···Cl1	0.98	2.92	3.576 (4)	125
C12—H12*B*···Cl1	0.98	2.98	3.636 (4)	125
C13—H13*B*···Cl1	0.99	2.85	3.560 (3)	130

Symmetry codes: (i) −*x*+1, −*y*+1, −*z*+1; (ii) *x*−1/2, −*y*+3/2, *z*+1/2.
